# Combined nano and micro structuring for enhanced radiative cooling and efficiency of photovoltaic cells

**DOI:** 10.1038/s41598-021-91061-1

**Published:** 2021-06-02

**Authors:** George Perrakis, Anna C. Tasolamprou, George Kenanakis, Eleftherios N. Economou, Stelios Tzortzakis, Maria Kafesaki

**Affiliations:** 1 Institute of Electronic Structure and Laser (IESL), Foundation for Research and Technology-Hellas (FORTH), 70013 Heraklion, Crete Greece; 2 Department of Materials Science and Technology, University of Crete, Heraklion, Crete Greece; 3 Department of Physics, University of Crete, 71003 Heraklion, Crete Greece; 4 Science Program, Texas A&M University at Qatar, P.O. Box 23874, Doha, Qatar

**Keywords:** Devices for energy harvesting, Solar cells, Solar cells, Optoelectronic devices and components

## Abstract

Outdoor devices comprising materials with mid-IR emissions at the atmospheric window (8–13 μm) achieve passive heat dissipation to outer space (~ − 270 °C), besides the atmosphere, being suitable for cooling applications. Recent studies have shown that the micro-scale photonic patterning of such materials further enhances their spectral emissivity. This approach is crucial, especially for daytime operation, where solar radiation often increases the device heat load. However, micro-scale patterning is often sub-optimal for other wavelengths besides 8–13 μm, limiting the devices’ efficiency. Here, we show that the superposition of properly designed in-plane nano- and micro-scaled periodic patterns results in enhanced device performance in the case of solar cell applications. We apply this idea in scalable, few-micron-thick, and simple single-material (glass) radiative coolers on top of simple-planar Si substrates, where we show an ~ 25.4% solar absorption enhancement, combined with a ~  ≤ 5.8 °C temperature reduction. Utilizing a coupled opto-electro-thermal modeling we evaluate our nano-micro-scale cooler also in the case of selected, highly-efficient Si-based photovoltaic architectures, where we achieve an efficiency enhancement of ~ 3.1%, which is 2.3 times higher compared to common anti-reflection layers, while the operating temperature of the device also decreases. Besides the enhanced performance of our nano-micro-scale cooler, our approach of superimposing double- or multi-periodic gratings is generic and suitable in all cases where the performance of a device depends on its response on more than one frequency bands.

## Introduction

Since the first demonstration of photonic daytime radiative cooling^1^, there is a continuously increasing interest in radiative cooling approaches and systems, as there is an increasing demand for even more efficient energy harvesting devices^2^. A radiative cooler may dissipate waste heat to the cold outer space (− 80 to − 270 °C) radiatively by emitting thermal radiation in the atmospheric transparency window at 8–13 μm, without consuming electricity^3^. This is an appealing approach to reduce global-energy consumption due to refrigeration^4^. Optoelectronic devices, such as solar cells, may also benefit from radiative cooling due to the negative temperature impact on their energy yield and lifetime^5–9^. Efficient radiative coolers for solar cell applications (i.e., employed as top coatings) must be transparent in the visible and near-infrared spectrum (at ~ 0.3 to 1.1 μm for silicon-based solar cells) and highly emissive in the thermal wavelengths (~ > 4 μm). Promising material candidates for the realization of such coolers are glasses, such as silica or low iron soda-lime glass; it is mainly because of their ability to act as optically transparent and fairly acceptable, blackbody-like, thermal radiators due to their phonon-polariton resonance modes at mid-infrared (mid-IR) wavelengths^10^. Moreover, such materials offer simplicity and reliability/stability compared, for example, to highly-emissive polymers, like polydimethylsiloxane (PDMS)^11^. Due to the increased thermal load in solar cell devices, studies aim to further enhance such materials' emissivity^12^, usually by applying a photonic patterning resulting in a surface structuring in the micro-scale. (Note that the energy radiated is directly proportional to the material's emissivity, which depends strongly on the geometry and the surface.) Examples of such patternings include silica micro-gratings composed of spherical particles^13^ and pillars^14^, pyramidal micro-texturing^15,16^, and two-dimensional silica photonic crystals^17^. Although most of the proposed structures exhibit an almost perfect blackbody emissivity, their implementation in the solar cell industry depends also on their fabrication feasibility and cost-effectiveness.

Several studies conclude that the key to achieve economically feasible radiative cooling is to pursue additional improvements^12^, e.g., to employ radiative coolers with enhanced transparency in the optical range and radiative cooling at the same time^18^. Apparently, manufacturing scalability is also an important factor. However, most radiative coolers are too thick (> 100 μm) with a micro-structuring that is often sub-optimal for other spectral regimes besides 8–13 μm, such as 0.3–1.1 μm, where a much smaller structuring size is required. Recently, Cho et al.^19^ proposed a scalable, on-chip, thin radiative cooler. To achieve high and broadband thermal emission, they fabricated hexagonally arrayed (8 μm pitch) holes (7 μm deep, 6 μm diameter), realized by submicron-thick (0.5/0.3 μm) SiO_2_/AlO_x_ double shells resulting to a hollow cavity film. When placed on top of a silicon wafer, the film exhibited a large emissivity of ~ 0.8 at omnidirectional incidence (0°–80°), as well as > 0.73 Si solar absorption ~ 19% enhancement compared to a bare Si wafer. Moreover, Long et al.^14^ proposed SiO_2_ pillar-micro-gratings with a simplified structure as solar-transparent and radiatively cooling thin coatings for solar cells. The well-designed grating, with a total thickness of 3.1 μm (2.3 μm pillar height), when placed on top of a doped silicon wafer, increased Si solar absorption by 111 W/m^2^ and enhanced emissivity in the atmospheric window compared to a flat SiO_2_ film. Besides the above structures, various promising designs have been proposed to enhance optical absorption, suitable for incorporation and efficiency enhancement in solar cells^20–22^. Still, though, it lacks a design/approach able to give optimum double-band operation (i.e., enhanced transparency in optical, enhanced absorption/emission in mid-IR), combined with a wide-angle response, in a thin, scalable and single-material structure. Here, we propose a double-periodic structure/approach aiming to fulfill all these requirements and, also, able to be combined with different structure-designs among the ones in the literature.

To achieve simultaneously high optical transparency and high thermal emission in a thin and scalable structure, essentially a metasurface, we superimpose properly designed in-plane nano- and micro-scaled periodic patterns on glass. An enhanced overall device performance (optical, thermal, electrical) arises from the combination of optimized-resonant surface nano- and microstructures for each spectral regime of interest (i.e., 0.3–1.1 μm and 8–13 μm, respectively). The combined efficiency gain is because the micro-grating's response in the mid-IR is not affected by the nano-grating (which controls the optical response) and vice versa. We apply this idea theoretically in the case of a scalable, few-micron-thick, and simple single-material (glass) radiative cooler. When we place the thin radiative cooling coating on top of a Si wafer, sunlight absorption in Si increases by ~ 25.4%. Such a performance is considerably larger than the achievable through the previously reported approaches. The emissivity in the mid-IR also improves compared to Si or a flat glass film. Temperature reduction and efficiency enhancement was also achieved when we applied the proposed cooler in realistic Si-based solar cells. Moreover, our proposed double periodic (or multi-periodic) approach for a photonic cooler design, besides leading to enhanced PV efficiencies, is scalable and can be successfully applied to any device affected by the management of photons of different/distant spectral regions. Additionally, it can be combined with different micro- or nano-grating patterns/shapes (e.g., pyramids, hemispheres) if different patterning appears more suitable depending on the applications, fabrication details or conditions. Thus, our proposed approach can pave the way for efficient multispectral and/or multifunctional metasurface coatings that can optimize the thermal, optical and electrical response of a system, targeting efficient optoelectronic devices and other electro-optical components.

## Nano-micro-grating on top of a Si wafer

**Figure 1:**
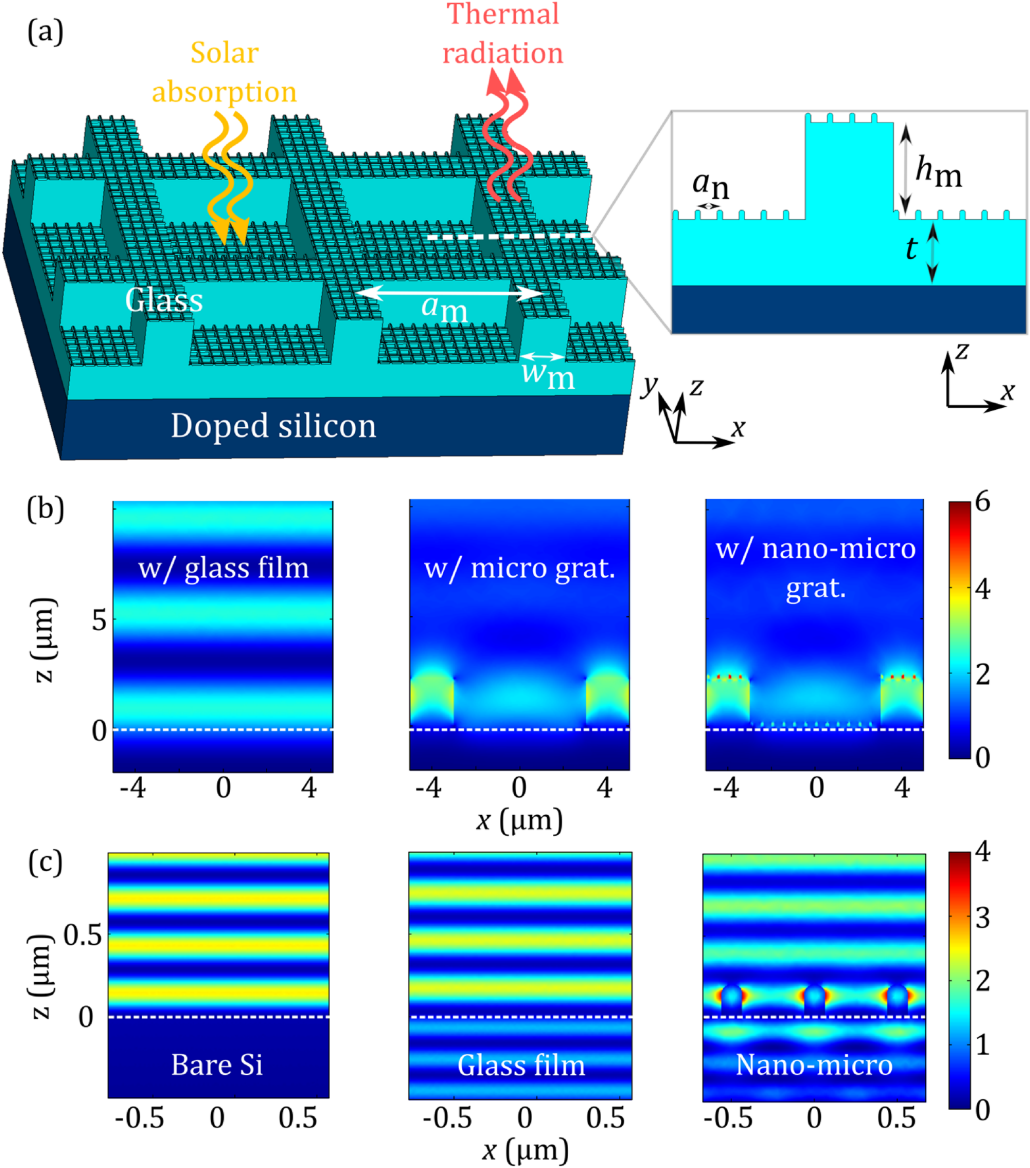
(**a**) Schematic of the nano-micro-structured glass grating coating atop of a doped Si layer, and a vertical cross-section of the micro-unit-cell. (**b**,**c**) Normalized (relative to the incident field, ***E***_0_) distribution of the squared amplitude of the electric field at *λ* = 8.7 μm, *θ* = 0° in (**b**) for a flat 3.7-μm-thick glass film on top of Si (left), the microstructured glass grating on top of Si (middle), and the nano-micro-structured glass grating on top of Si (right) cases, and *λ* = 0.58 μm, *θ* = 0° in (**c**) for the flat Si (left), flat 1.5-μm-thick glass film on top of Si (middle), and the nano-micro-structured glass grating on top of Si (right) cases. In (**c**) middle, we assume a 1.5-μm-thick glass film (instead of a deposited 3.7-μm-thick used as a reference in the present study) to demonstrate the impact of adding the nano-micro-grating on top.

In the first part of our studies we assume that our proposed thin glass nano-micro-structured cooling coating, which is a two-dimensional (2D—for achieving polarization insensitive response) subwavelength grating, is placed on top of a Si wafer (see Fig. 1a). For such a structure a variety of fabrication techniques are available^14,19,23–25^. The silicon wafer is assumed *p*-type heavily-doped with a dopant of boron and resistivity of 0.0013 Ω cm^26^. (This way we take into account the substantial emissivity of crystalline silicon solar cells in the mid-IR^14,27^.)

To obtain the structure’s thermal emissivity, we calculate the structure’s absorptivity in the mid-infrared wavelengths (~ > 4 μm) numerically. Absorptivity is defined as the absorbed fraction of incident radiation and can be obtained as *A* = 1 − *R*-*T*, where *R*, *T* are the reflectivity and the transmissivity, respectively. Following Kirchhoff’s law, we can directly determine the structure’s emissivity as it is equal to the absorption in the corresponding spectral regime under thermodynamic equilibrium. Calculations are performed using the commercial electromagnetic solver CST Studio. To achieve enhanced mid-IR emissivity, our grating is imprinted in soda-lime glass, a common encapsulant material in solar cell applications, with permittivity as given in^10^. The array period in the micro-scale is evaluated via numerical optimization targeting enhanced emissivity within the atmospheric transparency window at ~ 8 to 13 μm. [This regime also coincides with the spectral region where there is a strong impedance mismatch between glass and air (due to glass phonon-polariton resonance mode at ~ 8.4 to 11 μm^10^)]. The optimum array period was calculated to be *α*_m_ ≤ 8 μm. In our study, we set *α*_m_ = 8 μm, the highest optimum value, to be more convenient to accommodate experimentally the combined nano- and micro-scale structuring. The optimized microstructured grating has a pitch of *α*_m_ = 8 μm, a height of *h*_m_ = 2.2 μm, a width of *w*_m_ = 2 μm, and a thickness of *t* = 1.5 μm, as shown in Fig. 1a.

**Figure 2:**
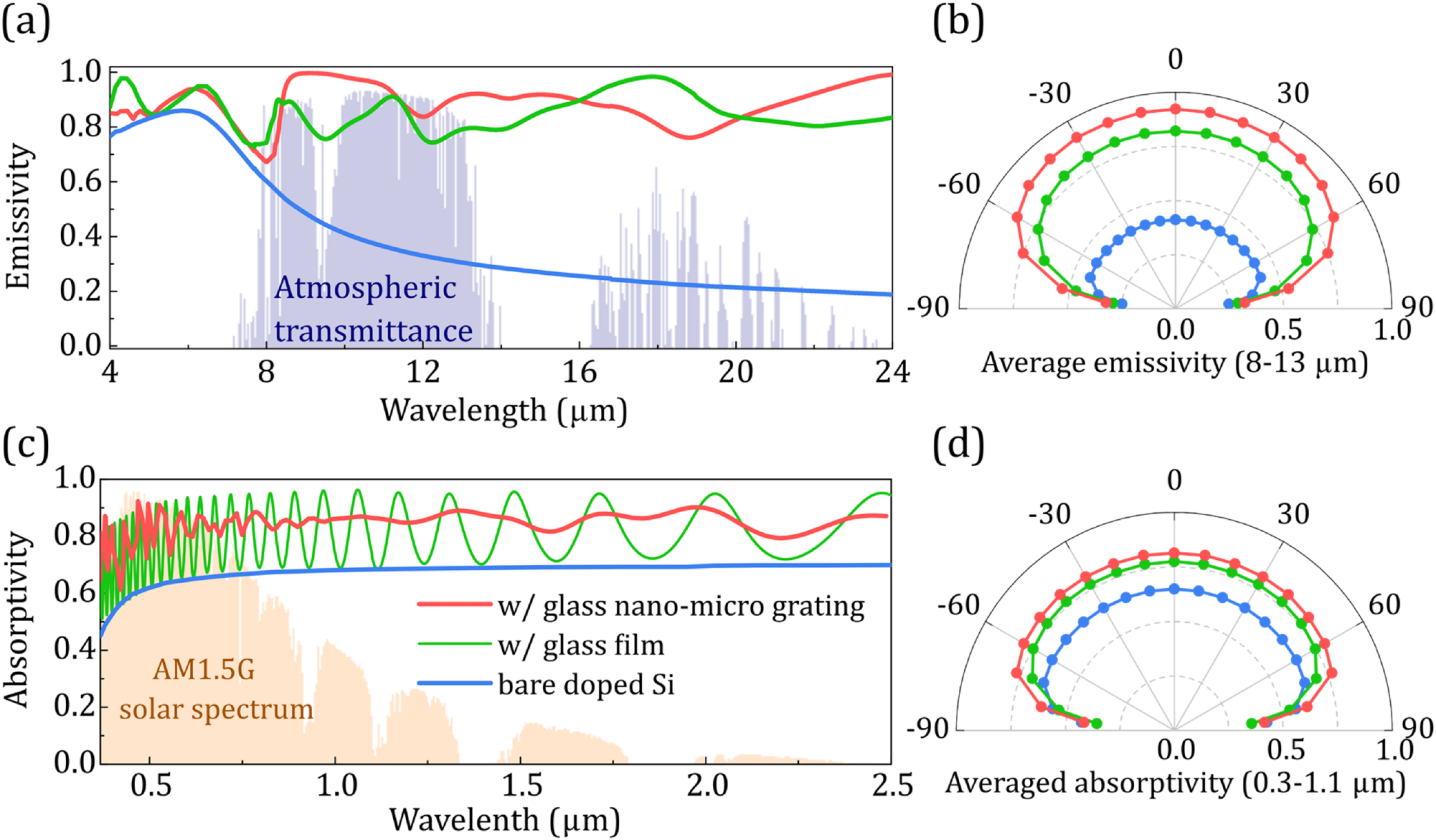
Absorptivity/emissivity spectra of the radiative cooler shown in Fig. 1a, in (**a**) thermal and (**c**) solar wavelengths, compared to a bare doped silicon and a Si covered by a 3.7-μm-thick glass film. (**b**) Angular dependence of the average emissivity at 8–13 μm (averaged over wavelength) for the systems shown in (**a**). (**d**) Absorbed fraction of solar radiation at 0.3–1.1 μm weighted by the AM1.5G spectrum^28^.

The resulting emissivity at the atmospheric transparency window (8–13 μm) is shown in Fig. 2a,b, where a significant enhancement compared to bare Si and a flat glass film is demonstrated (almost unity emissivity within the spectral region where there is a strong impedance mismatch between glass and air ~ 8.4 to 11 μm), which is maintained even for larger incidence angles (e.g. larger than 60°). This broad enhancement is related to the excitation of localized resonant modes in the pillars of the grating, for both *s*- and *p*-polarization, together with the lattice effect that excites collective in-plane surface states (see Fig. 1b). Such states arise from arrays satisfying the Bragg scattering condition, i.e., the array period α and the free space wavelength *λ*_0_ obey the equation *α*(√*ε* ± sin*θ*) = *l*λ_0_, where *ε* is the dielectric constant of the optical medium, *l* is an integer, and *θ* is the angle of incidence. Note that, by utilizing Kirchhoff’s law, we assume that the electromagnetic fields in the medium are in equilibrium with the matter (see Fig. 1b,c). This is valid even in non-equilibrium steady-state conditions (no net heat transfer) for the materials and systems examined^13,17^. Therefore, the enhanced field intensity in the medium results in enhanced structure's absorptivity/emissivity. This follows directly from Poynting’s theorem for power dissipation (*A*∼|***E***|^2^ where ***E*** is the electric field).

On top of the microstructured grating, and in order to achieve an enhanced transparency in the optical wavelengths, compared to a flat-glass film, we superimpose a two-dimensional nanostructured grating. To determine the optimum size of the nano-structuring, we perform an optimization analysis in the ranges: *h*_n_ = 0.1–0.65 μm, *ff* = 10–55%, and *α*_n_ = 0.1–0.65 μm, where *h*_n_ is the height, *ff* is the filling factor, and *α*_n_ is the pitch of the nano-pillars, respectively. (Such structures may be quite easily realized by laser induced nanostructuring^23,25,29,30^; therefore, their potential for light harvesting in solar cells is worth examining.) The resulting nano-structured grating has a pitch of *α*_n_ = 0.5 μm, a height of *h*_n_ = 0.21 μm, and a width of *w*_n_ = 0.13 μm. [Note that when the incoming wave impinges on the grating normally, utilizing high *α*_*n*_, i.e., ~  > 0.5 μm (higher than the diffraction limit, *α*_*n*_ > λ), higher diffraction orders become propagating within the wavelength range where the sun has its maximum intensity (~ 0.45 to 0.65 μm). This resulted in lower device solar absorption due to increased reflectivity.] The resulting broadband transparency (anti-reflectivity), compared to bare Si (see Fig. 2c,d), arises from two main effects. Firstly, the presence of the glass film (even flat glass) reduces the reflection at the air/Si interface. This is verified in Fig. 1c. This reduction comes from interferences within the thin film implied by the oscillations of the corresponding absorption curve (see Fig. 2c, green lines]^14^. Secondly, the resonant response of the nanostructured grating and the associated strong scattering result also to reduced reflection at the air/glass interface and thus to further absorption enhancement in Si (see Figs. 1c, 2c,d)^23,31,32^ (note that glass practically does not absorb at those wavelengths). We note that subwavelength resonant elements’ impact on the radiative properties of a device (reflection, transmission, absorption) can be also accurately described with various equivalent circuit model approaches^33,34^.

Comparing the ***E***-field intensity in Fig. 1b middle and right indicates that the impact of the micro-grating in mid-IR is not affected by the nano-grating validating the idea of superimposing nano-micro-structured gratings for enhancing device performance. Overall, the nano-micro-structured grating exhibits a high mid-IR emissivity of > 0.85 (at 0°–60°—see Fig. 2b) as well as > 0.79 (0°–60°) Si solar absorption (see Fig. 2d), yielding a ~ 25.4% solar absorption enhancement (at 0.3–1.1 μm), compared to a bare Si wafer, without increasing Si cell’s complexity. The Si absorption enhancement in the optical wavelengths provided by the proposed nano-micro-structured grating is considerably higher than that of the scalable thin radiative coolers proposed in the literature, with values of ~ 19.0% (compared, e.g., to the coolers proposed in^14,19^). To boost the absorptivity further, we can add atop the bare doped Si a thin (75 nm) index-matched Si_3_N_4_ layer (a typical example of an anti-reflection coating used in solar cells that increases cell’s solar absorption). When we place the nano-micro-grating coating on top of this structure, it results in a higher Si solar absorption of ~ 0.92%. The results of Fig. 2 indicate that the addition of a thin radiative cooling coating on top of a simple-planar bare doped Si cell results in a high solar absorptivity (with a wide-angle response) and high thermal emissivity, without increasing the complexity of the Si-based cell design. This approach could reduce the complexity and the cost of the cell technology concerning thin-film PV technologies where the simplicity of cell’s planar architecture should be maintained^22,35^ or in the case of concentrated PV systems.

From the manufacturing point of view, the proposed coating consists of relatively simple structures, i.e., simple gratings, which could be realized by laser-induced structuring. Specifically, besides the advancements in direct laser writing (DLW) and the formation of the well-known Laser-Induced Periodic Surface Structures (LIPSS)^23,29,30,36^, there has been recently a significant advancement in the field of Direct Laser Interference Patterning (DLIP)^36–40^. It relies on creating interference patterns by overlapping two or more laser beams and treating the material’s surface directly, which provides greater flexibility in the choice of target material and the surface texture, being suitable for the creation of deterministic double- or multi-periodic structures^36,37,39,40^. Due to the nature of laser-processing technology, the patterned surfaces can be easily scaled up to large areas that could be used to enhance the light-harvesting in solar cells. An additional advantage of our proposed double- or multi-periodic approach is that it could be realized with appropriate structures of different shapes (not only binary gratings). Moreover, the structure design (the meta-atoms) does not need to be of the same geometry in micro- and nano-scale. Appropriate nano-scale patternings on top of the micro-grating can facilitate the fabrication process significantly.

The enhanced emissivity provided by the nano-micro-grating at the atmospheric window implies improved device thermal response. To calculate the operating temperature (*T*) of the device, we employ a non-equilibrium steady-state power-balance, which is determined by summing the total power density “into” and “out of” the structure^17^. The power-balance equation can be expressed as

1 $${P}_{rad}\left(T\right)-{P}_{atm}\left({T}_{amb}\right)+{P}_{c}\left(T,{T}_{amb}\right)-{P}_{sun}=0,$$

where *P*_rad_ is the power radiated by the structure (mainly in the mid-IR, offering cooling to the device), *P*_atm_ is the power absorbed (by the structure) from the atmospheric emission, *P*_c_ is the power (absorbed or emitted) related to the non-radiative heat transfer, and *P*_sun_ is the absorbed solar power by the structure that dissipates into heat. They are given by (see, e.g., Ref.^17^ for details)

2 $${P}_{rad}\left(T\right)={\int }_{0}^{\infty }d\Omega cos\theta {\int }_{0}^{\infty }{\varphi }_{BB}\left(\lambda ,T\right)\varepsilon \left(\lambda ,\theta \right)d\lambda ,$$

3 $${P}_{atm}\left({T}_{amb}\right)={\int }_{0}^{\infty }d\Omega cos\theta {\int }_{0}^{\infty }{\varphi }_{BB}\left(\lambda ,{T}_{amb}\right)\varepsilon \left(\lambda ,\theta \right){\varepsilon }_{atm}\left(\lambda ,\theta \right)d\lambda ,$$

4 $${P}_{c}\left(T,{T}_{amb}\right)={h}_{c}\left(T-{T}_{amb}\right),$$

5 $${P}_{sun}={\int }_{0}^{\infty }{\varphi }_{AM1.5G}\left(\lambda \right)\varepsilon \left(\lambda ,{\theta }_{sun}\right)cos{\theta }_{sun}d\lambda ,$$

*T*_amb_ is the ambient temperature, *h*_c_ is the nonradiative heat transfer coefficient due to the convection and the conduction, *φ*_BB_(*λ*,*T*) is the spectral irradiance of a blackbody at temperature *T* given by Planck’s law, and *φ*_AM1.5G_(*λ*) is the solar illumination represented by the measured sun’s radiation, the AM1.5G spectrum^28^. Following Kirchhoff’s law, the spectral-directional absorptivity of the structure equals the spectral-directional emissivity, *ε*(*λ*,*θ*). ε_atm_(*λ*,*θ*) = 1–*t*(*λ*)^1/cos*θ*^ is the angle-dependent emissivity of the atmosphere, with *t*(*λ*) the atmospheric transmittance in the zenith direction^41^. *P*_rad_ and *P*_atm_ were considered within the range *λ* = 4–30 μm, while *P*_sun_ at *λ* = 0.28–4 μm.

**Figure 3:**
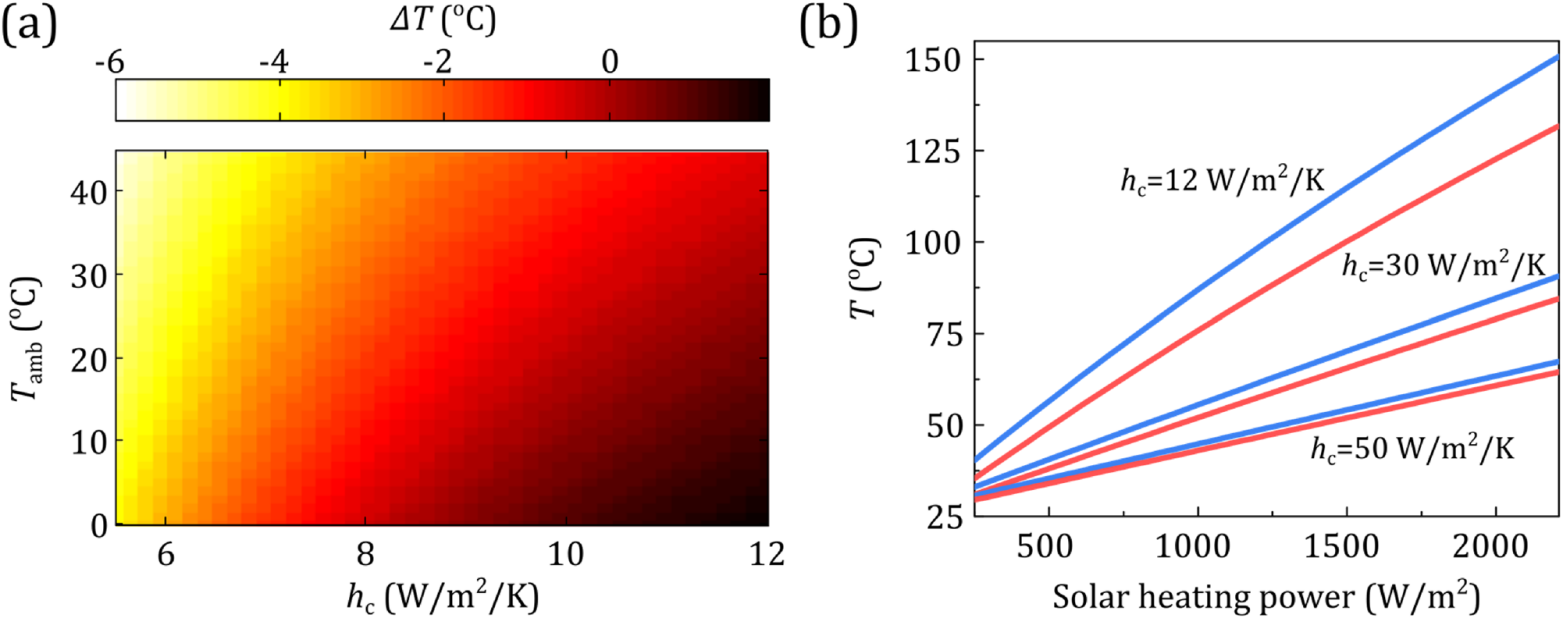
(**a**) Calculated steady-state temperature change, Δ*T *(°C), with the implementation of the nano-micro-structured grating on top of a bare doped silicon wafer, as a function of the conduction–convection coefficient, *h*_c_, and the ambient temperature, *T*_amb_. (**b**) Calculated steady-state temperature of Si with nano-micro-structured grating (red), and bare silicon (blue)] versus the solar heating power, for *h*_c_ = 12 W/m^2^/K (corresponding to natural convection) and 30, 50 W/m^2^/K (corresponding to forced convection or actively cooled systems).

The change in the bare Si wafer's steady-state temperature (Δ*Τ*) when we incorporate the glass nano-micro-grating radiative cooler is calculated and plotted in Fig. 3a. The nonradiative heat-transfer coefficient, *h*_c_, was taken in the range of 5.5–12 W/m^2^/K, corresponding to wind speeds of 1–3 m/s^14,19^, and the ambient temperature in the range of 0–44 °C.

The temperature decrease provided by the nano-micro-grating coating (of equivalent/average thickness 2.46 μm) compared to the bare doped silicon is approximately up to 5.8 °C, and up to 0.2 °C compared to the flat 3.7-μm-thick glass film case, despite the augmented solar absorption by 164.8 W/m^2^ and 36.1 W/m^2^, respectively, which is vital for achieving high photocurrents and therefore efficiencies. Indicatively, for silicon-based solar cells, a temperature decrease by 1 °C could increase the efficiency by ~ 0.45%_rel_, while the aging rate doubles if the temperature increases by 10 °C^8^. As shown in Fig. 3a, the positive impact of the nano-micro-grating coating on device temperature increases as outdoor temperature increases; it further increases (in a nonlinear manner) as *h*_c_ decreases, indicating the greater importance of radiative cooling in severe environmental conditions (e.g., high temperatures with low wind speeds), in particular when convective cooling is not adequate. We note that the positive temperature difference (Δ*T* > 0) at about *h*_c_ ~  > 10 W/m^2^/K is due to the reduced effectiveness of radiative cooling at higher *h*_c_ (higher wind speeds), since the excess heat is carried away by convection.

Another evaluation of our nano-micro-grating cooler is given in Fig. 3b, where we plot the device temperature in the case of the nano-micro-grating atop of doped silicon (red) compared to bare Si (blue), as a function of the solar heating power of the device, ranging from 250 to 2200 W/m^2^. (Note here that the solar heating power is considered the same in both systems, i.e., bare Si and Si with grating, unlike in Fig. 3a, where the increased heating power due to the cooler has been taken into account.) In this way we isolate the radiative cooing impact. Consequently, we roughly estimate the cooler impact also in concentrated PV systems (CPVs), where the absorbed solar power reaches values higher than 1000 W/m^2^. Indicatively, depending on the conditions (clouds, humidity), the expected heat output of a crystalline solar cell under peak unconcentrated solar irradiance (1000 W/m^2^) can reach values higher than 250 W/m^2^, up to approximately 730 W/m^2^. We observe that the temperature reduction, coming from the implementation of the radiative cooler, scales up almost linearly with the solar heating power. Even in the case of low-concentration systems and high *h*_c_, i.e., 30 and 50 W/m^2^/K, which correspond to actively cooled systems, the temperature reduction can reach values of ~ 6.2 °C and ~ 2.9 °C, respectively. Results in Fig. 3b demonstrate also the increased impact of passive radiative cooling in CPVs, due to their higher absorbed solar powers and high device temperatures that develop (> 80 °C)^27^. (Temperature reductions due to radiative cooling have been reported already in CPVs by using a conducting surface area larger than the cell itself^42^.) Despite their high operating temperatures, CPVs constitute promising candidates for efficient and cost-effective energy harvesting due to their high efficiencies, induced by incorporating low-cost concentrator lenses, and the associated lower electrical-power-temperature coefficients compared to conventional PVs. Our results demonstrate that radiative cooling can play a significant role in the case of CPVs, given that it can be employed efficiently in conjunction with other nonradiative cooling techniques, such as backside finned heat exchangers with/or forced air convection.

## Nano-micro-grating on top of a realistic PV device

**Figure 4:**
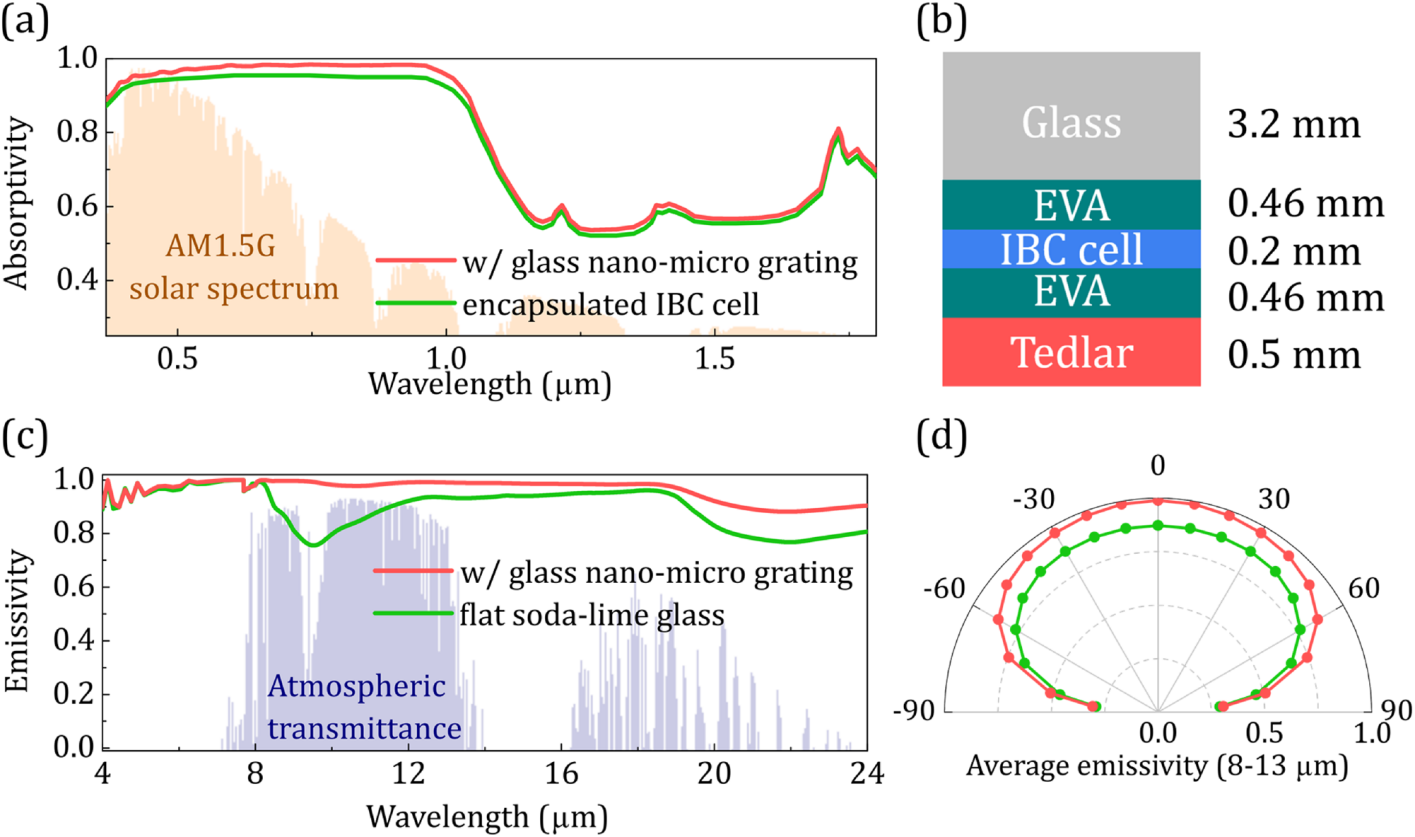
(**a**) Absorptivity spectra of the encapsulated IBC cell (green, see main text) and of the encapsulated cell with the glass nano-micro-grating on top of it (red). (**b**) Conventional material stacking of the encapsulated crystalline silicon-based IBC solar cell. (**c**) Emissivity spectra of a 3.2-mm-thick PV glass (green) and of the proposed nano-micro grating cooler assuming a semi-infinite glass substrate (red), and (**d**) angular dependence of the average emissivity at 8–13 μm.

Although several solar cell compatible radiative coolers have been proposed in the literature, most of them examine their impact on bare Si wafers and only a few studies exploit their impact on actual PV modules^5,11,42,43^, where the power-balance relation has to take into account also the transformation of part of the solar energy to electrical energy or its loss via other mechanisms^5,9^. Here we examine the impact of our nano-micro grating cooler on both the temperature and the efficiency of a realistic system, considering a crystalline silicon-based solar cell among the ones available in the market. The cell involves a mono-crystalline silicon wafer with interdigitated state-of-the-art type back contacts (IBC); the Si wafer is encapsulated within two 0.46 mm EVA (ethylene–vinyl acetate) layers (see Fig. 4b), while on the front there is a 3.2 mm glass and, on the rear, a 0.5-mm-thick Tedlar (polyvinyl fluoride) substrate.

The cell has an electrical-power-temperature coefficient − 0.29%/°C, the internal quantum efficiency (IQE) is obtained for an industrial grade IBC cell from^44^. We obtain the bare cell, encapsulated cell (Fig. 4a), and EVA absorptivity in the solar wavelengths from^5,27^, which contain experimentally measured values. Moreover, considering that the encapsulated cell thermal emissivity comes almost exclusively from its 3.2-mm-thick top glass layer, the material’s optical properties/data are obtained from^10^ for the simulations (see Fig. 4c,d) and correspond to low iron soda-lime glass with an averaged emissivity equal to ~ 0.85 (very close to a commercial solar glass ~ 0.84). Comparing the optical absorptivity and the mid-IR emissivity of the encapsulated cell without and with the nano-micro-grating cooler (see Fig. 4a,c,d), one can see already the high impact of the grating on the absorption and emission properties of the PV. This impact, as we show below, is translated to lower PV operating temperatures and enhanced efficiencies.

For calculating the operating temperature and efficiency for the above cell, we substituted the last term, *P*_sun_, in Eq. (1) by *P*_solar,heat_, obtained from

6 $${P}_{solar,heat}\left({V}_{mp},T\right)-{P}_{ele,mp}\left({V}_{mp},T\right)-{P}_{rad,cell}\left({V}_{mp},T\right)+{P}_{sun}=0,$$

where *V*_mp_ is the output voltage at the maximum power point (mp)^5^, *P*_ele,mp_ = − *J*_mp_*V*_mp_ is the maximum extracted electrical power, *J* is the current density, and *P*_rad,cell_(*V*_mp_,*T*) is the power density radiated by the cell through carrier recombination (also known as the non-thermal radiation) calculated according to the analysis we have thoroughly discussed in^5^, where we additionally incorporated IQE^44^ to accurately describe the solar to electrical power conversion efficiency. To take into account the power radiated by the rear Tedlar substrate, *P*_ground_, we also add in the power balance (Eq. 1) the term (given by Stefan–Boltzmann law)

7 $${P}_{ground}\left(T,{T}_{amb}\right)=\sigma {\varepsilon }_{Tedlar}A\left({T}^{4}-{T}_{amb}^{4}\right),$$

where *ε*_Tedlar_ = 0.85 is the hemispherical emissivity of Tedlar, *σ* is the Stefan–Boltzmann constant, and *A* ~ 1 is the view factor^18^. The power density loss due to convection owing to the air adjacent to the bottom and top surfaces is given by

8 $${P}_{c}\left(T,{T}_{amb}\right)={h}_{c}\left(T-{T}_{amb}\right)={h}_{c,top}\left(T-{T}_{amb}\right)+{h}_{c,bottom}\left(T-{T}_{amb}\right),$$

The resulting non-equilibrium steady-state power-balance is then given by

9 $${P}_{rad}\left(T\right)-{P}_{atm}\left({T}_{amb}\right)+{P}_{c}\left(T,{T}_{amb}\right)+{P}_{ground}\left(T,{T}_{amb}\right)-{P}_{solar,heat}\left({V}_{mp},T\right)=0,$$

while the cell efficiency, *η*, is calculated by the ratio of *P*_ele,mp_ to the total incident power.

**Figure 5:**
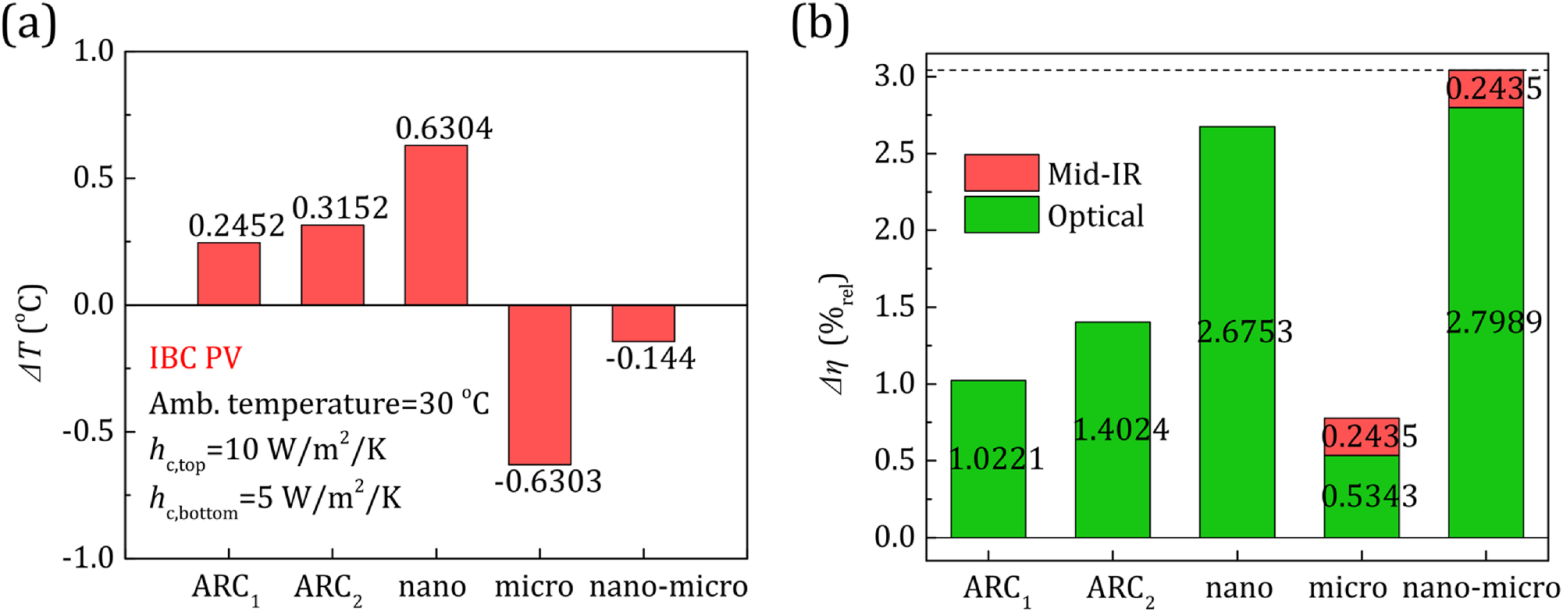
The change in the operating temperature (**a**) and efficiency (**b**) of the IBC PV of Fig. 4b resulting from the implementation of the only nano-, only micro- and nano-micro gratings of Fig. 1a, compared also to that of two conventional anti-reflection coatings (glass-ARCs). The parameters shown in the legends are explained in the main text.

In Fig. 5, we depict the impact of the glass nano-grating, the glass micro-grating, and the glass nano-micro-grating when applied on the encapsulated IBC cell (Fig. 4b), on the operating temperature and efficiency change (comparing to the conventional cell—with only the flat-glass). Additionally, we compare with two common glass anti-reflection coatings (glass ARCs), which are a 99 nm porous SiO_2_ layer (ARC_1_) and a thin low-index MgF_2_ layer (ARC_2_), with data extracted from^45^. The PV is assumed to operate under the following environment conditions (from^11^): illumination under the AM1.5G spectrum (1000 W/m^2^), *T*_amb_ = 30 °C, *h*_c,top_ = 10 W/m^2^/K (~ 1.2 m/s wind speed), *h*_c,bottom_ = 5 W/m^2^/K (~ 0.8 m/s wind speed).

As seen in Fig. 5a, the operating temperature increases with the implementation of ARC_1_, ARC_2_, and glass nano-grating. This temperature increase is due to the enhanced optical absorption associated with the increased heat dissipation within the Si absorption band range (0.28–1.107 μm), often called thermalization losses, as well as the increased unwanted absorption at the sub-bandgap range (1.107–4 μm). On the other hand, the glass micro-grating results in a temperature reduction of ~ 0.6 °C due to the improved thermal response in the thermal wavelength range in the mid-IR. Interestingly, the implementation of the combined nano-micro-grating still results in an operating temperature reduction (~ 0.1 °C) rather than an increase, despite the increased optical absorption (at 0.28–4 μm—red line in Fig. 4a). (Note that if one assumes zero optical absorption enhancement, the temperature reduction would be slightly higher.)

In Fig. 5b, we plot the relative efficiency increase (gain), Δ*η*(%_rel_), with the implementation of each radiative cooler. Namely, we calculate the optical gain, Δ*η*_optical_(%_rel_), assuming conventional (flat glass) PV emissivity in the thermal wavelengths in the mid-IR. Accordingly, we calculate the gain due to the emissivity enhancement in the mid-IR, Δ*η*_mid-IR_(%_rel_), by assuming flat glass absorptivity in the optical. For the total gain, Δ*η*_total_(%_rel_), we take into account both the optical and the mid-IR responses from each cooler. As seen in Fig. 5b, substantial optical gains arise for the cases of the glass nano- and nano-micro-structured gratings, considerably higher than those of conventional glass ARCs. Indicatively, the glass nano- and the glass nano-micro-structured grating achieve optical gains of ~ 2.8% compared to ~ 1.0% and ~ 1.4% of ARC_1_ and ARC_2_, respectively. Moreover, the glass nano-micro-grating provides additional gain due to the enhanced emissivity in the mid-IR (see Fig. 4c,d), resulting to total gain of ~ 3.1%. Examining different environmental conditions (different *h*_*c*_ and *T*_amb_), we found that the efficiency increase, Δ*η*(%_rel_), compared to the conventional case, is always higher than 2.96%.

## Conclusions

We have demonstrated that the superposition of properly designed in-plane nano- and micro-scaled periodic patterns in a thin glass radiative cooler on top of a Si-based solar cell can significantly enhance solar cells’ performance. The microscale patterning enhances cooler’s performance in the atmospheric transparency window (8–13 μm) while the nanopatterning further enhances the optical radiation absorption (i.e., in the Si absorption band: 0.28–1.1 μm). The combined enhancement is possible because the emissivity enhancement provided by the micro-grating in the thermal wavelengths is not affected by the nano-grating and vice versa. We evaluated the impact of the proposed nano-micro-structured grating on the temperature and absorption efficiency of a commercial PV cell and we found a relative efficiency increase higher than 3.0%, performance surpassing any previously reported results in the literature. Thus, our findings can significantly impact the PV industry, as well as other radiative cooling applications. Moreover, our proposed combined double- or multi-periodic patterning approach is a simple, single-material and scalable approach, suitable for all devices affected by photon management at different wavelength ranges/scales.
